# Gettysburg Katalysine Water

**Published:** 1868-11-01

**Authors:** 


### *Gettysburg Katalysine Water.*

Our attention has been called to the general interest on the subject of Mineral Spring Waters, and while we do not accept the many extravagant statements of interested parties respecting the medicinal value of these newly discovered springs, we regard the subject as eminently worthy the attention of medical men, aside from the claims put forth by those actuated mainly by pecuniary motives. There is ample evidence given upon high professional authority, of the demonstrated curative powers known to exist in many of the mineral waters in this country, and a great number in different places on the continent of Europe.

These latter have, in some instances, such as the Baden Baden, Vichy, and others, as well as our own Saratoga Springs, attained a reputation by long continued use and application to many diseases not always as successfully treated by medicines artificially compounded according to the best theories and practice known to the profession.

We should do injustice to the real claims of these natural fountains of healing waters, if we omit to say that our conviction is, that much of the prevalent ignorance on this subject among even the most scientific men in the medical profession, is the result of a prejudice on account of the empirical modes adopted to influence the purchase and sale of mineral waters.

In these remarks, we wish to be understood as referring only to *natural waters*, in distinction from artificial waters manufactured, as is pretended, by formulæ derived from analyses of natural springs. Such productions are, at best, a mere approximation to the original, and the best writers do not admit the possibility of any artificial combination which can exhibit the demonstrated therapeutic agency which resides in the natural combination, since the most delicate chemical tests fail to detect the subtle elements which are the real secret of the peculiar success not unfrequently attained by the intelligent and proper application of mineral waters.

Statements which come to us on good authority, respecting the medicinal properties of the Gettysburg Katalysine Water, as shown by many and repeated trials, seem clearly to indicate that this Water possesses remarkable curative powers, especially in cases of uric and lithic acid conditions, resulting in rheumatism, gout, and diseases of the renal apparatus generally, and its effects in these cases, as well as in dyspepsia and general prostration of the vital powers of the system, are such as to claim the attention of those whose complaints, as above, indicate its application as a remedy. While this Water is almost tasteless, like ordinary spring water, its effects, in cases of irritation of the kidneys and bladder, extending through the urethra, and even chronic affections of this nature, frequently yield by the use only of a gill of the Water at a time, and this three times a day. The discharges become less acrid, and the incipient calculi and uric formations are rapidly solved, and pass off in quantities according to the accumulations present.

All that the proprietors of this Water ask or claim, is the use of the Water, daily, as above suggested, for a sufficient length of time to control the acid tendency, and thereby restore the mucous surfaces to their normal condition, meanwhile, it is stated, the superior curative powers of the Water will be demonstrated to the satisfaction of both invalid and physician.

It appears a fortunate peculiarity that from its peculiar composition, this Water is unaffected by climate or exposure.

As we learn that many of the medical profession are testing the Water in this city, we shall be pleased to hear more of its effects in treatment generally.

---

A variety of editorial matter, items, etc., unavoidably crowded out of the present number.

